# A Simulated Investigation of Lithium Niobate Orientation Effects on Standing Acoustic Waves

**DOI:** 10.3390/s23198317

**Published:** 2023-10-08

**Authors:** Ranjith D. Janardhana, Nathan Jackson

**Affiliations:** 1 Department of Mechanical Engineering, University of New Mexico, Albuquerque, NM 87131, USA; 2 Nanoscience and Microsystems Engineering, University of New Mexico, Albuquerque, NM 87131, USA

**Keywords:** standing acoustic waves, acoustofluidics, cell separation, microelectromechanical systems, FEM, SAW

## Abstract

The integration of high-frequency acoustic waves with microfluidics has been gaining popularity as a method of separating cells/particles. A standing surface acoustic wave (sSAW) device produces constructive interference of the stationary waves, demonstrating an increase in cell separating efficiency without damaging/altering the cell structure. The performance of an sSAW device depends on the applied input signal, design of the IDT, and piezoelectric properties of the substrate. This work analyzes the characteristics of a validated 3D finite element model (FEM) of LiNbO_3_ and the effect on the displacement components of the mechanical waves under the influence of sSAWs by considering XY-, YX-, and 128^0^ YX-cut LiNbO_3_ with varying electrode length design. We demonstrated that device performance can be enhanced by the interference of multiple waves under a combination of input signals. The results suggest that 128^0^ YX-cut LiNbO_3_ is suitable for generating higher-amplitude out-of-plane waves which can improve the effectiveness of acoustofluidics-based cell separation. Additionally, the findings showed that the length of the electrode impacts the formation of the wavefront significantly.

## 1. Introduction

Acoustofluidics is an emerging field of research that combines acoustics with microfluidics. Acoustofluidics can be used in a wide range of applications, including particle separation, micro-mixing, channel sorting, bubble formation, and atomization, all of which involve particle or fluid manipulation due to acoustic waves [1]. The common application of acoustofluidics is in the areas of cell sorting, particle separation, fluid mixing, and atomization and involves the creation of out-of-plane surface waves using a surface acoustic wave (SAW) device that interacts with fluid media. There have been significant advances in using acoustofluidics in particle separation and in understanding the mechanism of action, which includes energy transmission from acoustic waves, flow of the liquid, wave transmission through microfluidic channels, and acoustic radiation force. These high-propagating mechanical waves are a combination of planar and transverse waves that operate at higher frequencies (100 s of MHz) and decay along the depth of the piezoelectric substrate [2,3,4,5]. The frequency of the waves is dependent on the acoustic velocity of the piezoelectric material and wavelength which is determined by the width and spacing of the interdigitated transducers (IDTs) or electrodes. Basic SAW devices are relatively easy to fabricate as they consist of a piezoelectric substrate and an electrically conducting IDT. More complicated structures could include coatings, reflectors, and phononic crystals to guide the waves. A voltage applied to the IDT produces a traveling SAW (tSAW) through the piezoelectric material. Due to the anisotropic features of the piezoelectric material, the wave formation is dependent on the crystal cut of the material, which affects the acoustic velocity and amplitude components [6].

When SAW devices are integrated with a microfluidic channel consisting of a liquid medium with cells or particles, the propagating waves can be used to separate particles based on size. According to the cell properties such as size, density, and compressibility, the acoustofluidics-based cell separation is the only effective contactless technique that can separate cells without altering or damaging the cells [7,8]. The suspended particles in the microfluidic channel undergo separation due to the predominance of the acoustic radiation force as well as acoustic streaming acting on the particles. The performance of these acoustofluidics methods depends heavily on the performance of the SAW device, being influenced by key factors such as wavefront propagation, operating frequency, displacement components of waves, and IDT design [9,10,11]. Recent experimental research work on standing SAWs (sSAWs) has demonstrated an improvement in particle/cell separation efficiency [12,13,14,15,16]. Numerical simulation analysis illustrated that the interference of the standing wave increased the out-of-plane amplitude of the waves, which improved the separation of cells/particles due to an increase in acoustic energy. Also, the length of the IDT affects the cell separation [8,17,18,19,20]. Therefore, increasing the acoustic energy of the SAW could result in enhanced particle separation capabilities.

Despite extensive experimental work establishing the phenomena, the modeling and simulation of acoustofluidics regarding cell/particle sorting are limited. There has been minimal research conducted by considering the high aspect ratio of length to width of the IDT to investigate the effects of SAW design based on performance. In this regard, this paper explores the characteristics and interference of stationary waves of sSAW devices and considers the higher aspect ratio of length to width of a LiNbO_3_ IDT using a finite element model (FEM) in COMSOL Multiphysics commercial software [21]. The goal of this work is to facilitate the acoustic energy along the microfluidic channel by increasing the out-of-plane displacement of the waves by considering the longer length of the IDT design and sSAWs. Initially, the FEM of three-dimensional (3D) X-cut Y-propagating LiNbO_3_ consisting of an IDT length of 4500 µm was validated against experimental and simulation results based on the performance factor “Insertion Loss” (IL). The effect of parameters such as the thickness of the IDT and impulse properties were studied. After validating the FEM model, this study further investigates the characteristics of the X-cut Y-propagating LiNbO_3_ 3D model. This article also analyzes the impact of sSAWs on stationary wave amplitude under different input signal conditions using three different cuts of LiNbO_3_: 3D XY LiNbO_3_, 3D YX LiNbO_3_, and 3D 128^0^ YX LiNbO_3_. We also investigated the displacement characteristics caused by combining multiple waves. The FEM developed could be useful for researchers interested in enhancing the performance of acoustofluidic devices and could be integrated into a more complex model involving microfluidics in the future.

## 2. Materials and Methods

### 2.1. Model Equation

The constitutive equation for a piezoelectric material that relates electric field and strain [22] is given by

(1) $$T_{ij}=C_{ijkl}\;S_{kl}-e_{kij}E_{k},$$

(2) $$D_{i}=e_{ikl}\;S_{kl}+\epsilon_{ik}E_{k}.$$

where *T* refers to the stress tensor, *S* indicates the strain tensor, $S_{ij}=\frac{1}{2}\left(\frac{\partial u_{i}}{\partial x_{j}}+\frac{\partial u_{j}}{\partial x_{i}}\right)$, *e* is the piezoelectric constant, and *D* is electric displacement. The elastic stiffness matrix, electrical field vector, and permittivity matrix are indicated by *C*, *E,* and *ϵ* respectively.

The wave equation [23] in the piezoelectric material is expressed as

(3) $$\frac{\partial T_{ij}}{\partial x_{j}}=\rho \frac{\partial^{2}u_{i}}{\partial t^{2}}\;.$$

where ρ refers to the density of mass and $u_{i}$ are displacement components. The electric field was approximated as quasi-static, and the electric charge was considered to be zero [23,24]. Maxwell’s equation becomes

(4) $$E_{i}=-\frac{\partial V}{\partial x_{i}},$$

(5) $$\frac{\partial D_{i}}{\partial x_{i}}=0$$

where *V* indicates electric potential. Further, the piezoelectric constitutive relation is reduced to coupled wave equations by solving Equations (1)–(5) as follows,

(6) $$-\rho \frac{\partial^{2}u_{i}}{\partial t^{2}}+C_{ijkl}\;\frac{\partial^{2}u_{k}}{\partial x_{j}\partial x_{l}}+e_{kij}\frac{\partial^{2}V}{\partial x_{k}\partial x_{j}}=0,$$

(7) $$e_{ikl}\;\frac{\partial^{2}u_{k}}{\partial x_{i}\partial x_{l}}-\epsilon_{ik}\frac{\partial^{2}V}{\partial x_{i}\partial x_{k}}=0.$$

with three degrees of freedom for displacement ($u_{i}$) and one for electric potential (*V*). The convergence error was calculated using *L*_2_ norm [25] on the domain Ω = (a, b) and given by

(8) $${\left\|u-u_{h}\right\|}_{L_{2}}={\left(\int_{a}^{b}{\left|u-u_{h}\right|}^{2}\;dx\right)}^{\frac{1}{2}}\;.$$

where *u* is considered as reference coarse mesh and *u_h_* is a finer mesh. *h* stands for the length of an element.

### 2.2. Computational Domain

#### 2.2.1. Two-Dimensional Domain

**Figure 1:**
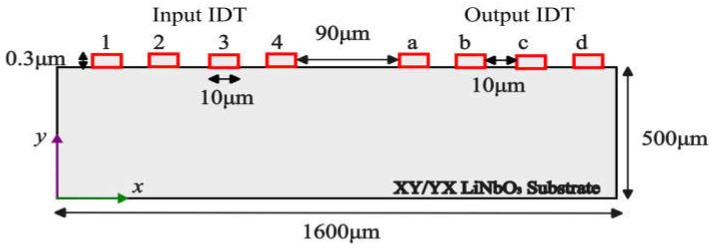
Two-dimensional computational domain for SAW device.

The 2D computational model of XY LiNbO_3_ used for the verification study is represented in Figure 1. A LiNbO_3_ substrate with dimensions of 1600 µm × 500 µm was used in the initial study. The input and output ports consisted of two-finger pairs of aluminum IDTs with dimensions of 0.3 µm thick and 10 µm wide with a pitch of 10 µm. The input and output IDTs were named “1”, “2”, “3”, and “4” as well as “a”, “b”, “c”, and “d”. The chosen periodicity of IDTs was 10 µm (λ/4) where the wavelength (λ) was equal to 40 µm. The active area for the microfluidic channel was between the input and output IDTs with a width of 90 µm. The aforementioned computational domain was based on the previously published report [26].

#### 2.2.2. Three-Dimensional Domain

**Figure 2:**
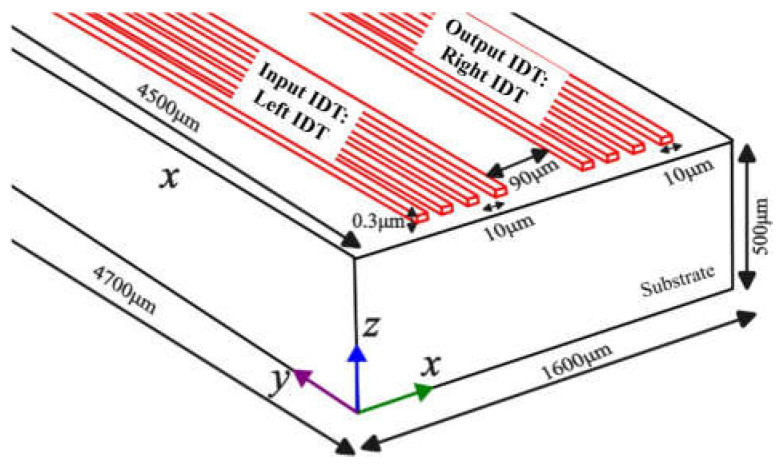
Three-dimensional computational domain for SAW device.

The 3D computational domain chosen was similar to the 2D domain with overall dimensions 1600 µm × 500 µm × 4700 µm as depicted in Figure 2. The longer length of the electrode of 4500 µm was selected, with a thickness and width of 0.3 µm and 10 µm, respectively. The reason for selecting a higher length-to-width aspect ratio was to study the wave formation and reduce the amplitude along the length of the electrode, along with creating a near 50 Ω resistance needed for impedance matching. Here the input IDT and output IDT are also referred to as the left and right IDTs, respectively. All other dimensions remained identical to the 2D domain. The total active area under the influence of acoustic waves was 90 µm × 4500 µm.

#### 2.2.3. Mesh and Boundary Conditions

**Figure 3:**
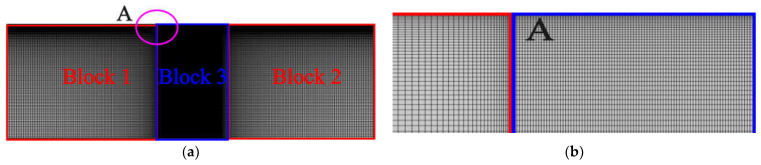
Mesh for the (**a**) 2D SAW device; (**b**) zoomed view of area “A” near IDT.

An example of the 2D computational mesh with a zoomed view of area “A” close to IDT is shown in Figure 3a,b. The finer mesh was applied near the surface of the IDT to capture the surface wave utilizing quadrilateral mesh. Also, the finer mesh was used in a region, “block 3”, which consists of active area and IDT as represented in Figure 3. The 2D mesh details are provided in Table 1. A similar finer mesh was utilized for the 3D domain by keeping 40 elements per wavelength near the IDT.

The left and right sides of the 2D domain were enforced with periodic boundary conditions whereas the bottom of the domain was kept fixed. All four sides were applied with periodic boundary conditions, and the bottom part was applied with fixed conditions in the case of the 3D domain. The following impulse signal ($V_{i}$) was applied at the input IDT as an initial condition for the verification and validation study. The impulse function was applied to the IDT fingers “2” and “4”, and “1” and “3” (Figure 1) were set to ground.

Step Pulse [27]:

(9) $$V_{i}=\left\{\begin{array}{c} \;1\;V,\;0\leq t\leq 1\;ns.\\ 0,\;t\geq 1\;ns. \end{array}\;\right.\;$$

A peak-to-peak sinusoidal voltage of 1 *V* with a frequency of 100 MHz was applied at the input IDT for characteristic analysis of 3D XY LiNbO_3_ (section: 3D model). To determine open circuit voltage, the output IDT was configured for the floating potential condition [27]. However, for sSAW case studies, the input signal was applied to both the left and right IDTs (Figure 2) to investigate the effects of combining waves. Table 2 lists the material properties of the IDTs.

#### 2.2.4. Crystal Orientation

Due to the anisotropic characteristics of LiNbO_3_, different orientations of the cut with respect to the crystallographic axes affect material properties such as the elastic stiffness matrix (*C*), piezoelectric constant (*e*), acoustic velocity, and permittivity matrix (*ϵ*). In COMSOL Multiphysics, crystal cut was applied by rotating the coordinate system based on Euler angles. COMSOL Multiphysics documentation [28] contains further detailed explanations for implementing the material orientation. XY-cut LiNbO_3_ indicates that the surface wave propagates in the Y direction normal to the X and Z surface [29]. Correspondingly, YX-cut LiNbO_3_ refers to the surface wave generated in the X direction that was normal to the Y and Z direction. Euler angle was set to (0, −90, −90) for XY cut LiNbO_3_ for rotating the coordinate system in COMSOL whereas the (0, 90, 0) was applied for YX cut LiNbO_3_. 128^0^ Y cut X propagation Euler angle was set to (0, −38, 0) [7] in COMSOL. Crystal orientation for the LiNbO_3_ cuts is depicted in the appendix section (Appendix A).

#### 2.2.5. Numerical Simulation

Numerical simulation was set up according to a previous study [11] that extended the simulation to a 3D model case. COMSOL Multiphysics was used for solving the wave equation (Equations (6) and (7)). The frequency response of the system was analyzed using time-dependent simulation with a time step of 0.1 *ns* with a total simulation time of 100 *ns*. This time dependent simulation parameters were similar to previous investigations [11]. Step pulse ($V_{i}$) mentioned in Equation (9) was applied at the input IDT and the impulse response of the device was recorded at the output IDT. Fast Fourier Transform (FFT) [26] was implemented to acquire the SAW device’s frequency response by using “Time to Frequency FFT” option in COMSOL. IL was determined using the following expression [30].

(10) $$IL=20log\left|\frac{V_{output}}{V_{i}}\right|\;$$

In contrast, the shape order of the displacement variables was set as quadratic serendipity while electric potential as quadratic to reduce computational time. The default values of COMSOL were used for other solver parameters.

## 3. Results and Discussion

### 3.1. Validation of a Model

**Figure 4:**
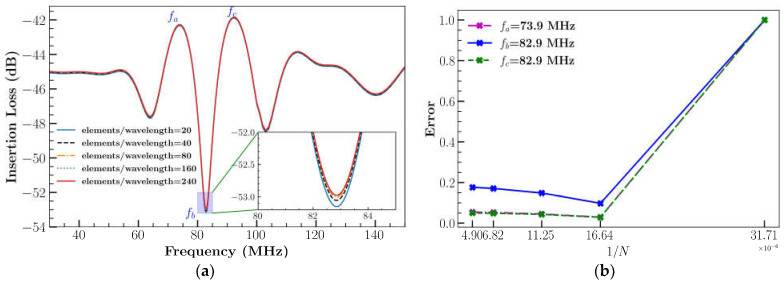
(**a**) Mesh sensitivity analysis; (**b**) error measured for 2D XY LiNbO_3_ using different total number of elements.

IL is calculated by using Equation (10) with time step = 0.1 *ns* with a total simulation time of 100 ns and impulse condition from Equation (9). The results are represented in Figure 4a for different cases (Table 1). The normalized error was calculated for 2D XY LiNbO_3_ at various frequencies using the L_2_ norm (Equation (8)) with respect to different total number of elements as shown in Figure 4b.

The findings are shown in Figure 4a,b. The error decreases with finer mesh and absolute errors were found to be within 1% between case 2 and case 5. Case 2 was opted for the remaining simulations due to decreased computational requirements with similar error.

**Figure 5:**
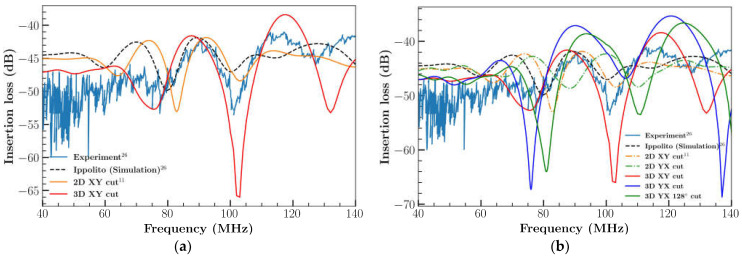
(**a**) Comparison of IL with experimental and simulation results; (**b**) comparison of IL with experimental and simulation results with different orientation of LiNbO_3_.

**Figure 6:**
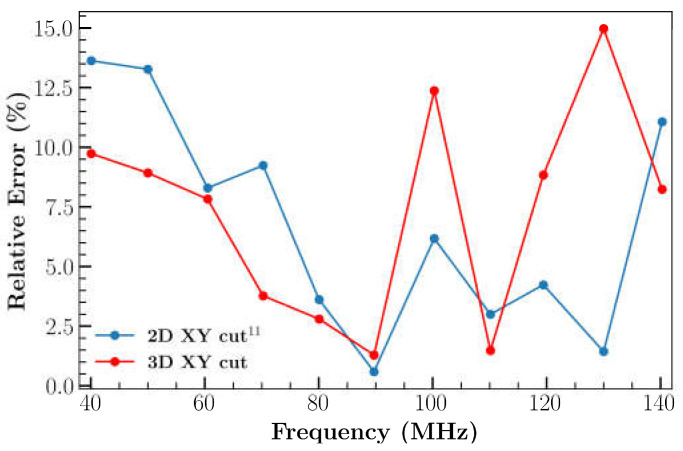
Comparison of relative error between experimental result and simulation result for 2D and 3D XY cut.

Further FEM analysis was extended to 3D cases with different orientations and cuts: 3D XY LiNbO_3_, 3D YX LiNbO_3_ and 3D YX 128^0^ LiNbO_3_. The results are illustrated in Figure 5a,b. Compared to the 2D XY cut, the 3D XY cuts of LiNbO_3_ results were in good agreement with the experimental outcomes [26] (Figure 5a). The addition of the extra dimension (length) to the model resulted in enhanced IL, which is in good agreement with previous results [31]. However, results of IL differ at higher frequency. This could possibly be caused by the number of elements considered for IDT, the time step, the diffracted waves produced by IDT boundaries due to available computational resource limitation. This demonstrates that the wave formation in the third direction influences the device’s performance. Figure 5b represents the comparison of simulation and experimental results of different orientations of LiNbO_3_. 3D XY cut LiNbO_3_ model captures a better result than a 2D model in most of the frequency locations as represented in relative error comparison plot Figure 6 with the experimental result.

### 3.2. Characteristics of Parameters

#### 3.2.1. Thickness

**Figure 7:**
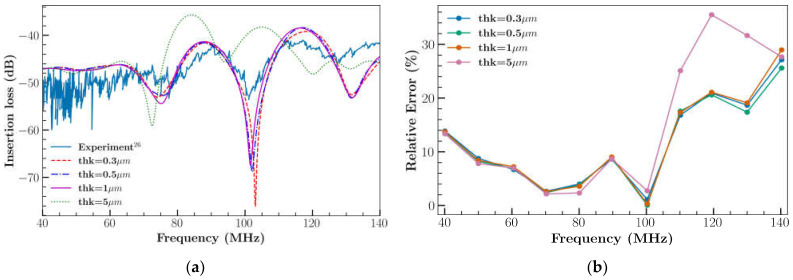
(**a**) Effect of electrode thickness on IL; (**b**) comparison of relative error between experimental result and simulation result by varying the thickness of electrode.

A parametric study was conducted to investigate the impact of electrode thickness on IL by utilizing the 3D XY LiNbO_3_ model. The impact of the electrode thickness on IL was represented in Figure 7a. A lower frequency shift was observed due to the increment in the thickness of the electrode. In addition, Figure 7b illustrates the relative error of IL. A significant increase in IL was determined at frequencies > 100 MHz for thick (5 μm) electrodes, which is believed to be due to the increased mass. A 0.5 μm electrode thickness was selected for all future simulations due to the reduced error.

#### 3.2.2. 3D Model

**Figure 8:**
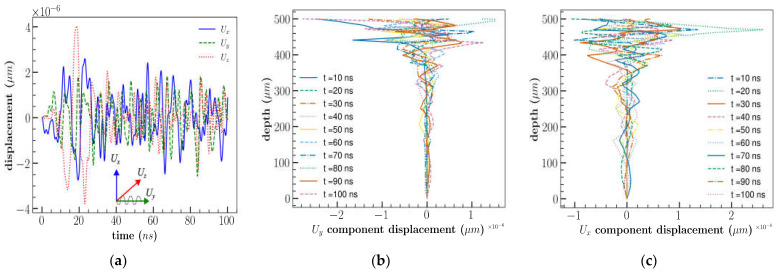
Simulation results of 3D XY LiNbO_3_ (**a**) displacement components with respect to time, at location (*x* = 800 µm, *y* = 200 µm, *z* = 500 µm); (**b**) *U_y_*; (**c**) *U_x_* displacement component along the depth at location (*x* = 800 µm, *y* = 200 µm).

The characteristics of the 3D XY LiNbO_3_ model were analyzed using the impulse condition mentioned in Equation (9). Figure 8a shows the displacement components of 3D XY LiNbO_3_ at location (*x* = 800 µm, *y* = 200 µm, *z* = 500 µm, Figure 2) for a duration of 100 *ns*. Results illustrate the effect of displacement in the *U_z_* direction, which is normal to the propagation direction (*U_y_*). IL (Figure 5) was affected by the displacement of the *U_z_* component which resulted in a good agreement with the predicted IL for the 3D XY LiNbO_3_. The amplitude of both *U_x_* and *U_y_* components were predominant at the surface and decayed along the depth of 6–8 wavelength of the device as represented in Figure 8b,c. Similar simulation outcomes for 3D model SAW devices were reported in previous studies [26,30]. Hence later simulation analysis was performed by considering the 3D model of LiNbO_3_. Contour plots of deformation for 3D XY LiNbO_3_ at different time steps are provided in the appendix section (Appendix A).

### 3.3. SAW

#### 3.3.1. Single Direction Travelling SAW

**Figure 9:**
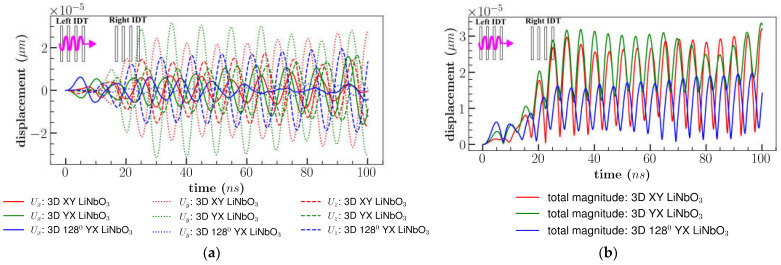
Comparison of (**a**) displacement components; (**b**) total displacement magnitude for different orientation of LiNbO_3_ SAW for single direction.

Different orientations of the 3D model of LiNbO_3_: (a) XY cut, (b) YX cut and 128^0^ YX cut were subjected to the peak-to-peak continuous sinusoidal voltage of 1 *V* and frequency of 100 MHz applied at left IDT only (Figure 2). This subsection was referred to as single direction or traveling SAW (tSAW). IL and displacement components (perpendicular to propagation direction) were analyzed in the material coordinate system (X, Y, Z) and represented in Figure 9a,b at the same location (800 µm, 200 µm, 500 µm).

**Figure 10:**
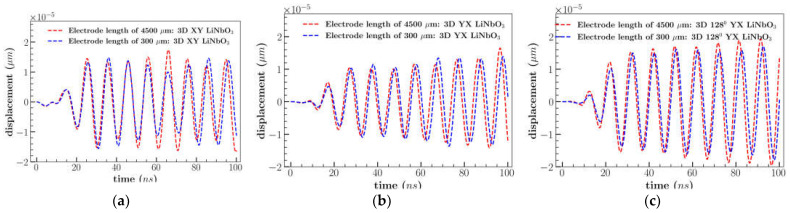
Comparison of *U_z_* displacement for (**a**) XY; (**b**) YX; (**c**) 128^0^ YX cut LiNbO_3_ of electrode length of 4500 µm and 300 µm.

Figure 9a indicates that the *U_y_* displacement component was predominant for XY and YX cut LiNbO_3_ with order of magnitude ∼10^−5^ µm and least for 128^0^ YX cut LiNbO_3_ (∼10^−6^ µm). The out-of-plane displacement (*U_z_*) which was normal to the propagation direction was found to be maximum for 128^0^ YX cut LiNbO_3_. The variation in the dominance of the displacement components was due to the anisotropic property of the materials under different cuts. The impact of varying the length of the electrode on *U_z_* component was insignificant as shown in Figure 10. However, *U_x_* displacement component was decreased to an average of 50% for XY cut and increased to 50% for YX cut in comparison to the design consisting of electrode length 300 µm. Similar to XY cut, displacement component *U_x_* was reduced for 128^0^ YX cut with electrode length of 4500 µm. It may be noted that the length of the electrode affects the displacement component along *U_x_* and similar results were observed in previous studies which improved the cell separation due to the longer length of the electrode [8]. A detailed comparison of displacement components for varying electrode lengths is provided in the appendix section (Appendix A).

**Figure 11:**
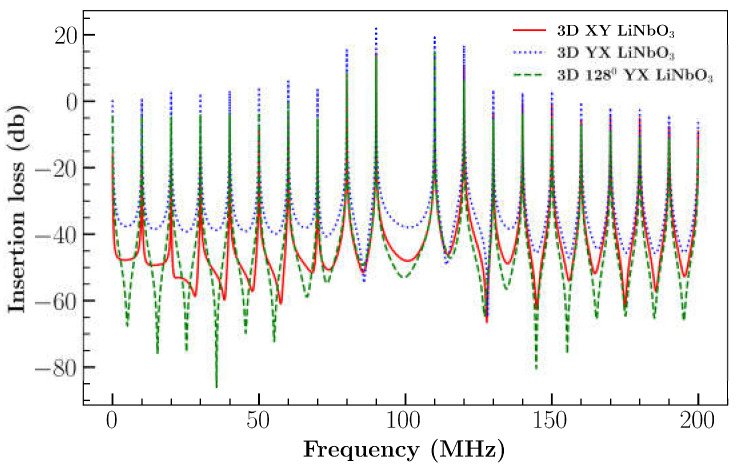
Comparison of IL for different orientations of LiNbO_3_ for single direction.

The total magnitude was highest for YX cut (average of 3 × 10^−5^ µm) due to the large displacement of the *U_y_* component (Figure 9b). On the other hand, the 128^0^ YX cut had the lowest total magnitude because of the low *U_y_* component but 128^0^ YX had the largest *U_z_* displacement with an average peak of 1.8 × 10^−5^ µm (Figure 10c). Figure 11 represents the comparison of IL between LiNbO_3_ orientation. IL was found to be minimum for YX cut with a range from 22 dB to −66 dB in comparison with YX cut 128^0^ LiNbO_3_ (range: 14 to −86 dB) and XY cut LiNbO_3_ (range: 14 to −65 dB).

#### 3.3.2. Bi-Directional/s SAW

**Figure 12:**
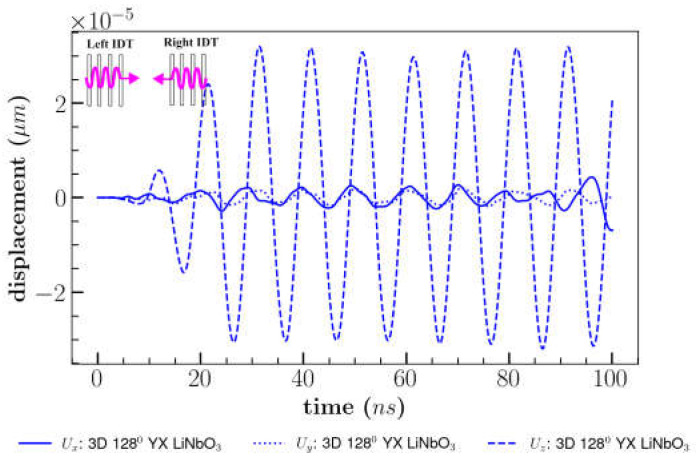
Comparison of displacements for 128^0^ YX cut LiNbO_3_ combining multiple waves.

This section demonstrates the effect of applying the same peak-to-peak voltage to both sets of IDTs working in both directions (left and right IDT Figure 2) and visualizes the effect of interference amplitude. The vertical displacement (*U_z_*) of 128^0^ YX cut LiNbO_3_ was increased by 65–70% compared to single direction due to interference of the stationary waves as shown in Figure 12. According to the result, *U_x_* displacement was reduced to 50% when compared to single direction case by establishing dominance in the out-of-plane direction for 128^0^ YX cut LiNbO_3_. Similar effects were reported in previous experimental investigation, demonstrating the formation of maximum out-of-plane amplitude for 128^0^ YX cut LiNbO_3_, which resulted in enhanced cell separation efficiency [15].

**Figure 13:**
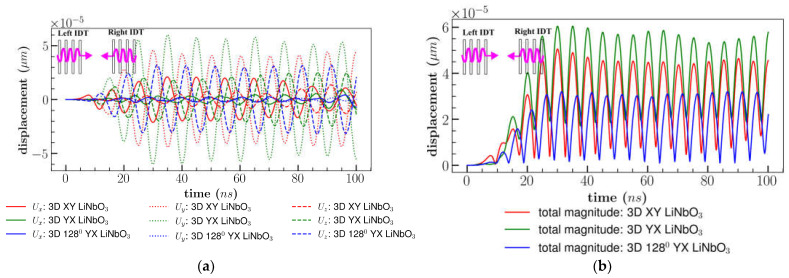
Comparison of (**a**) displacement components; (**b**) total displacement magnitude for different orientation of LiNbO_3_ combining multiple waves.

Figure 13a,b represent the displacement components under sSAW for different orientations of LiNbO_3_. For both XY and YX cut LiNbO_3_, predominant displacement component *U_y_* increased due to the constructive interference of the stationary waves. However, the amplitude of the *U_z_* component reduced to 50% for XY cut which resembles the shear waves for XY cut LiNbO_3_. In contradiction, *U*_x_ component decreased for YX cut. Due to an increase in the amplitude of the dominant component, the total displacement magnitude became larger for all cuts.

#### 3.3.3. Bi-Directional Wave Delay

We investigated the inference effects of stationary waves under the influence of peak-to-peak voltage by creating two sets of waves in opposite directions. Here, the input continuous signal was applied to the left IDT from the beginning, however, the right IDT was delayed by 50 *ns*. For comparison between the results of the earlier subsection, the delay input signal to the right IDT was turned on at the halfway point of the total simulation time to demonstrate its impact.

**Figure 14:**
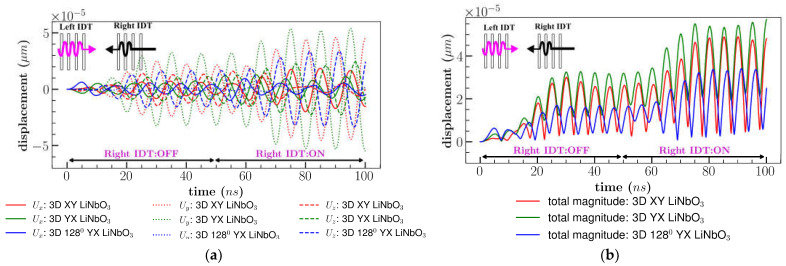
Comparison of (**a**) displacement components; (**b**) total displacement magnitude for different orientation of LiNbO3 for bi-direction (wave delay).

Results are depicted in Figure 14a,b by comparing the different orientations of the LiNbO_3_. The total displacement magnitude for all cuts were similar to a single input until 50 *ns*. However, delaying the right IDT, increased the amplitude of XY cut by an average of 5% and 128^0^ YX cut by 6% after 80 *ns*, nonetheless, results of YX cut remain unaltered in comparison to the bi-directional condition as represented in Figure 14b. Constructive waves generated in the active area (microfluidic channel area) did not reach a stable displacement until 30 *ns* after right IDT activation, but delaying the input signal enhanced the interference amplitude which led to improving the amplitude of the overall displacement after 80 *ns*.

**Figure 15:**
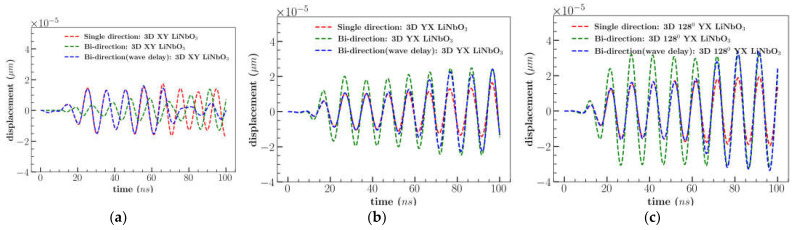
Simulation results of out-of-plane (*U_z_*) displacement considering cases single direction, bi-direction and bi-direction (wave delay) for (**a**) XY, (**b**) YX, and (**c**) 128^0^ YX cut LiNbO_3_.

Figure 15 represents the overall comparison for out-of-plane (*U_z_*) displacement of three cuts for three input signal cases: single direction, bi-direction, and bi-direction (wave delay). The maximum amplitude (*U_z_*) was observed to be higher for 128^0^ YX cut in all three-input signal conditions by 50% in comparison to other cuts. Combining the multiple waves improved the out-of-plane (*U_z_*) displacement for 128^0^ YX cut. However, delaying the right IDT increased the *U_z_* displacement marginally by 6% after 80 *ns*. A series of plots are provided in the appendix section (Appendix A) which compares the individual displacement component for the three cases: single direction, bi-direction, and bi-direction (wave delay).

Overall, the 3D FEM model of XY LiNbO_3_ performed well with experimental findings [26] in comparison to the 2D model based on the parameter IL. The characteristic investigation demonstrated that high frequency propagation waves were restricted to the surface and the thickness of the electrode affects the operating frequency of the device. According to the results of numerical analysis for single direction case, the higher significant displacement was observed in the length direction *U_y_* (according to material coordinate system, Figure 2) for XY and YX cuts, whereas out-of-plane amplitude (*U_z_*) was predominated for 128^0^ YX cut. The length of the electrode reduced the magnitude of *U_x_* component to 30–60% for XY and 128^0^ YX cut while it increased for YX cut in comparison with a shorter electrode (electrode length 300 µm). This suggests that the length of electrode affects the other displacement components and also previous studies specified that the efficiency of cell separation based on acoustofluidics depends on the length of the electrode [8].

The combination of the multiple input signal (bi-direction) conditions increased the displacement of the predominant components for all cuts. Due to the constructive interference of the stationary waves the out-of-plane (*U_z_*) component was increased to 50% for 128^0^ YX cut and a significant reduction in the displacement of the *U_x_* and *U_y_* component (Figure 15c). Similar observations were demonstrated in earlier studies which improved the cell separation efficiency by sSAW [15]. Wave formation in the XY cut was comparable to shear waves (inplane) with a considerable decrease in the magnitude of the *U_z_* component by the interference of the waves. Delaying the input signal marginally improved the amplitude of the dominating component in XY and 128^0^ YX cut whereas YX cut remained unchanged. However, the particle/cell separation efficiency of the device also depends on various aspects such as IDT design, width of the formed surface waves, microfluidic channel material and acoustic energy. Other important factors that impact cell separation are acoustic radiation and drag force induced by the acoustic streaming on the suspended particles. Cell separation due to acoustic radiation is mostly determined by particle dimensions and wavelength. The size of the particles affects both forces and previous studies [32,33] demonstrated that their relative dominance leads to the separation of the particles. The effect of these forces under the sSAW could be investigated in future work. 

## 4. Conclusions

The current work compared 2D and 3D FEM models of XY-cut LiNbO_3_ results with experimental data [26]. Numerical analysis findings on the high aspect ratio of length to width of IDT imply that the *U_x_* component of the mechanical waves depends on the IDT length. Three orientations of LiNbO_3_: XY cut, YX cut and 128^0^ YX cut were analyzed under different input signal conditions (i.e., single, bi-direction and bi-direction (wave delay)) by applying a peak-to-peak voltage to the left and right IDTs. Single-direction input signal results showed that the predominance of surface waves amplitude was dependent on crystal cut. Conversely, the coherence of the multiple waves improved the predominant wave displacement by 50% reducing the other displacement components under bi-direction and wave delay conditions. Wave delay study shows that the combination of applying continuous and pulse signal requires matching of the pressure antinode and node for the enhanced displacement. Hence, device performance can be improved by adjusting the displacement components which results in altering the interference amplitude of the waves through bi-direction/sSAW. From the analysis, it was observed that 128^0^ YX cut LiNbO_3_ were able to produce a higher amplitude of out-of-plane waves which would be most suitable for cell separation application based on acoustofluidics. This base FEM model can be integrated with microfluidics considering the other influential factors in subsequent work, and experiments would be conducted to further validate the model.

## Figures and Tables

**Table 1:** Mesh details for 2D domain.

	Total Number of Elements (*N*)	Number of Elements Per Wavelength (*λ*)
Case 1	31,540	20
Case 2	60,080	40
Case 3	88,910	80
Case 4	146,570	160
Case 5	204,230	240

**Table 2:** Material properties of IDTs.

Property	Aluminum (IDT)
Young’s Modulus (kPa)	70 × 10^9^
Poisson’s ratio	0.33
Density (kg/m3)	2700

**Figure A1:**
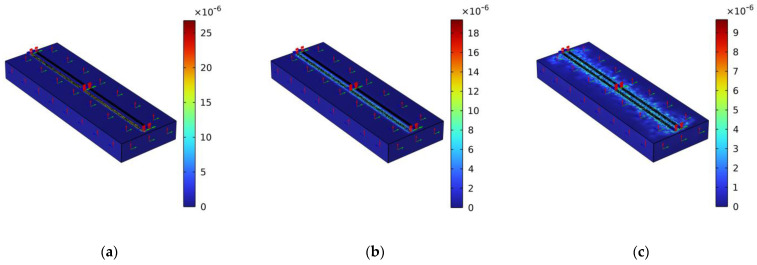
Simulation results of 3D XY LiNbO_3_ on (**a**) t = 1 ns; (**b**) t = 10 ns; (**c**) t = 100 ns.

**Figure A2:**
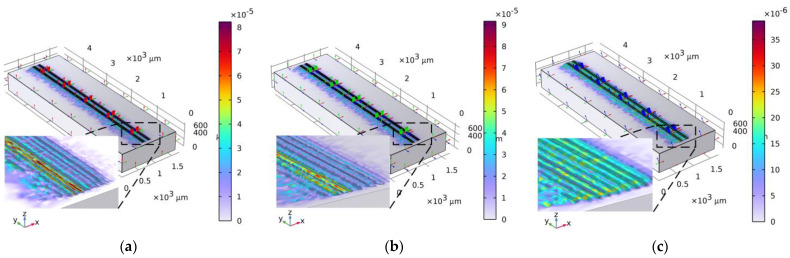
Contour plot of deformation with crystal orientations for (**a**) XY; (**b**) YX; (**c**) 128^0^ YX cut LiNbO_3_ single direction.

**Figure A3:**
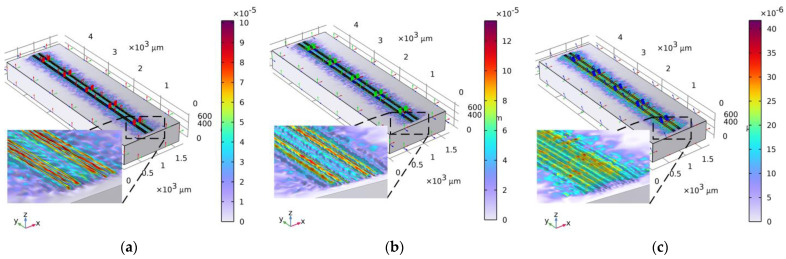
Contour plot of deformation with crystal orientations for (**a**) XY; (**b**) YX; (**c**) 128^0^ YX cut LiNbO_3_: Bi-direction.

**Figure A4:**
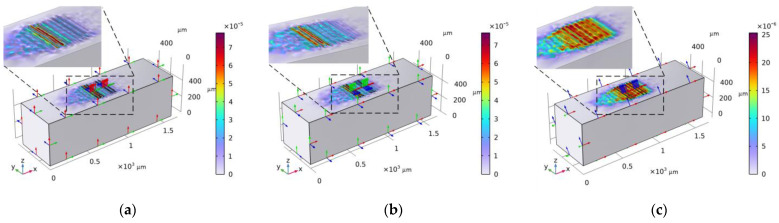
Contour plot of deformation with crystal orientations for (**a**) XY; (**b**) YX; (**c**) 128^0^ YX cut LiNbO_3_: Bi direction.

**Figure A5:**
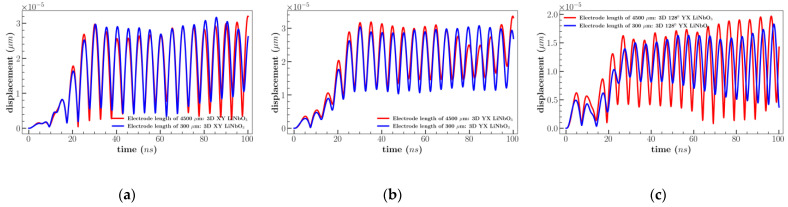
Simulation results of total displacement for (**a**) XY; (**b**) YX; (**c**) 128^0^ YX cut LiNbO_3_.

**Figure A6:**
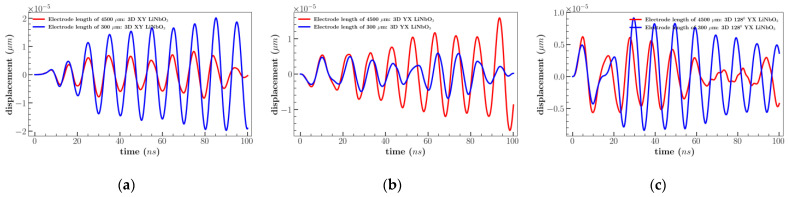
Simulation results for *U_x_* displacement for (**a**) XY; (**b**) YX; (**c**) 128^0^ YX cut LiNbO_3_.

**Figure A7:**
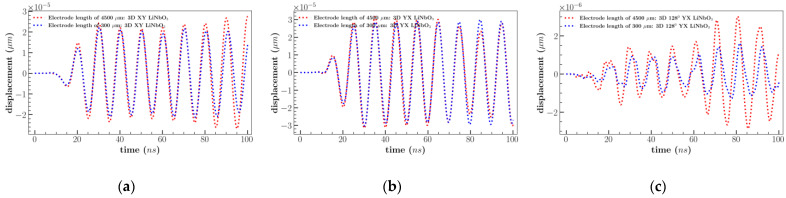
Simulation results for *U_y_* displacement for (**a**) XY; (**b**) YX; (**c**) 128^0^ YX cut LiNbO_3_.

**Figure A8:**
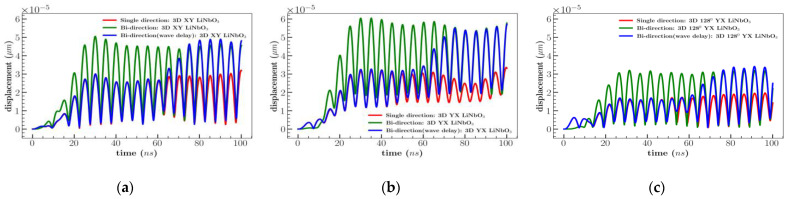
Simulation results of for total displacement for (**a**) XY; (**b**) YX; (**c**) 128^0^ YX cut LiNbO_3_.

**Figure A9:**
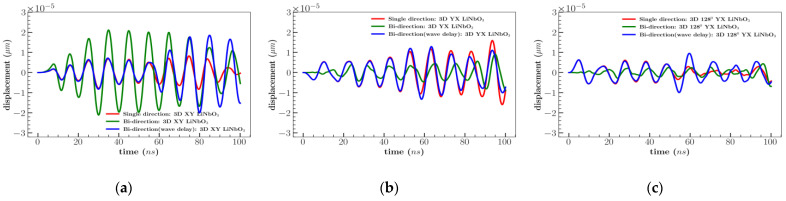
Simulation results for *U_x_* displacement for (**a**) XY; (**b**) YX; (**c**) 128^0^ YX cut LiNbO_3_.

**Figure A10:**
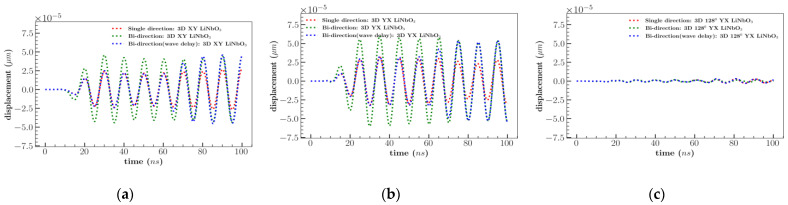
Simulation results for *U_y_* displacement for (**a**) XY; (**b**) YX; (**c**) 128^0^ YX cut LiNbO_3_.

## Data Availability

Not applicable.

## Appendix A. Additional Simulation Results

### Appendix A.4. Deformation Contour for Electrode Length 300 µm: Single Direction

The computational domain for shorter length electrode was chosen with overall dimensions 1600 µm × 500 µm × 500 µm. The length of the electrode was considered as 300 µm (shorter) with thickness and width of 0.5 µm and 10 µm respectively.
